# Immune checkpoint inhibitors and pericardial disease: a systematic review

**DOI:** 10.1186/s40959-024-00234-0

**Published:** 2024-05-17

**Authors:** Sarah E. Mudra, Danny L. Rayes, Ankit Agrawal, Ashwin K. Kumar, Jason Z. Li, Meredith Njus, Kevin McGowan, Kazi A. Kalam, Charalompos Charalampous, Mary Schleicher, Muhammad Majid, Alvena Syed, Abdullah Yesilyaprak, Allan L. Klein

**Affiliations:** 1 Department of Internal Medicine, MedStar Georgetown University Hospital, MedStar Health, Washington, DC USA; 2 Center for the Diagnosis and Treatment of Pericardial Diseases, Section of Cardiovascular Imaging, Department of Cardiovascular Medicine, Heart, Vascular, and Thoracic Institute, Cleveland Clinic, 9500 Euclid Ave., Desk J1-5, Cleveland, OH 44195 USA; 3 Floyd D. Loop Memorial Library, Cleveland Clinic, Cleveland, OH USA

**Keywords:** Immune Checkpoint inhibitors, Pericarditis, Pericardial Effusion, Cardiac Tamponade

## Abstract

**Introduction:**

Despite the growing use of immune checkpoint inhibitors (ICI) in cancer treatment, data regarding ICI-associated pericardial disease are primarily derived from case reports and case series. ICI related pericardial disease can be difficult to diagnose and is associated with significant morbidity. We conducted a systematic review to further characterize the epidemiology, clinical presentation, and outcomes of this patient population.

**Methods:**

A search of four databases resulted in 31 studies meeting inclusion criteria. Patients > 18 years old who presented with ICI mediated pericardial disease were included. Intervention was medical + surgical therapy and outcomes were development of cardiac tamponade, morbidity, and mortality.

**Results:**

Thirty- eight patients across 31 cases were included. Patients were majority male (72%) with a median age of 63. Common symptoms included dyspnea (59%) and chest pain (32%), with 41% presenting with cardiac tamponade. Lung cancer (81%) was the most prevalent, and nivolumab (61%) and pembrolizumab (34%) were the most used ICIs. Pericardiocentesis was performed in 68% of patients, and 92% experienced symptom improvement upon ICI cessation. Overall mortality was 16%.

**Discussion:**

This study provides the most comprehensive analysis of ICI-mediated pericardial disease to date. Patients affected were most commonly male with lung cancer treated with either Nivolumab or Pembrolizumab. Diagnosis may be challenging in the setting of occult presentation with normal EKG and physical exam as well as delayed onset from therapy initiation. ICI-associated pericardial disease demonstrates high morbidity and mortality, as evidenced by a majority of patients requiring pericardiocentesis.

**Supplementary Information:**

The online version contains supplementary material available at 10.1186/s40959-024-00234-0.

## Introduction

Immune checkpoint inhibitors (ICIs) have revolutionized cancer treatment, but they are associated with a range of immune-related adverse events (irAEs), including pericardial disease [1]. Despite the growing use of ICIs, data regarding ICI-associated pericardial disease are primarily derived from case reports and case series [2]. 

The exact mechanism of irAEs is incompletely understood currently. Recent studies suggest that both cardiac muscle and tumor cells have several high frequency T-cell receptor sequences in common suggesting a shared antigen theory [3]. However, others suggest that the development of myopericarditis may be due to underlying predisposing conditions that increase risk of development of disease [3]. 

Overall, given that there are no clear guidelines for diagnosis and treatment, and that delayed diagnosis portends an increased risk of mortality, further understanding of this complicated phenomenon is needed. Therefore, given the relative increase in ICI usage, we attempted to ascertain updated epidemiologic, clinical, and outcomes related data regarding this patient population.

## Methods

**Fig. 1:**
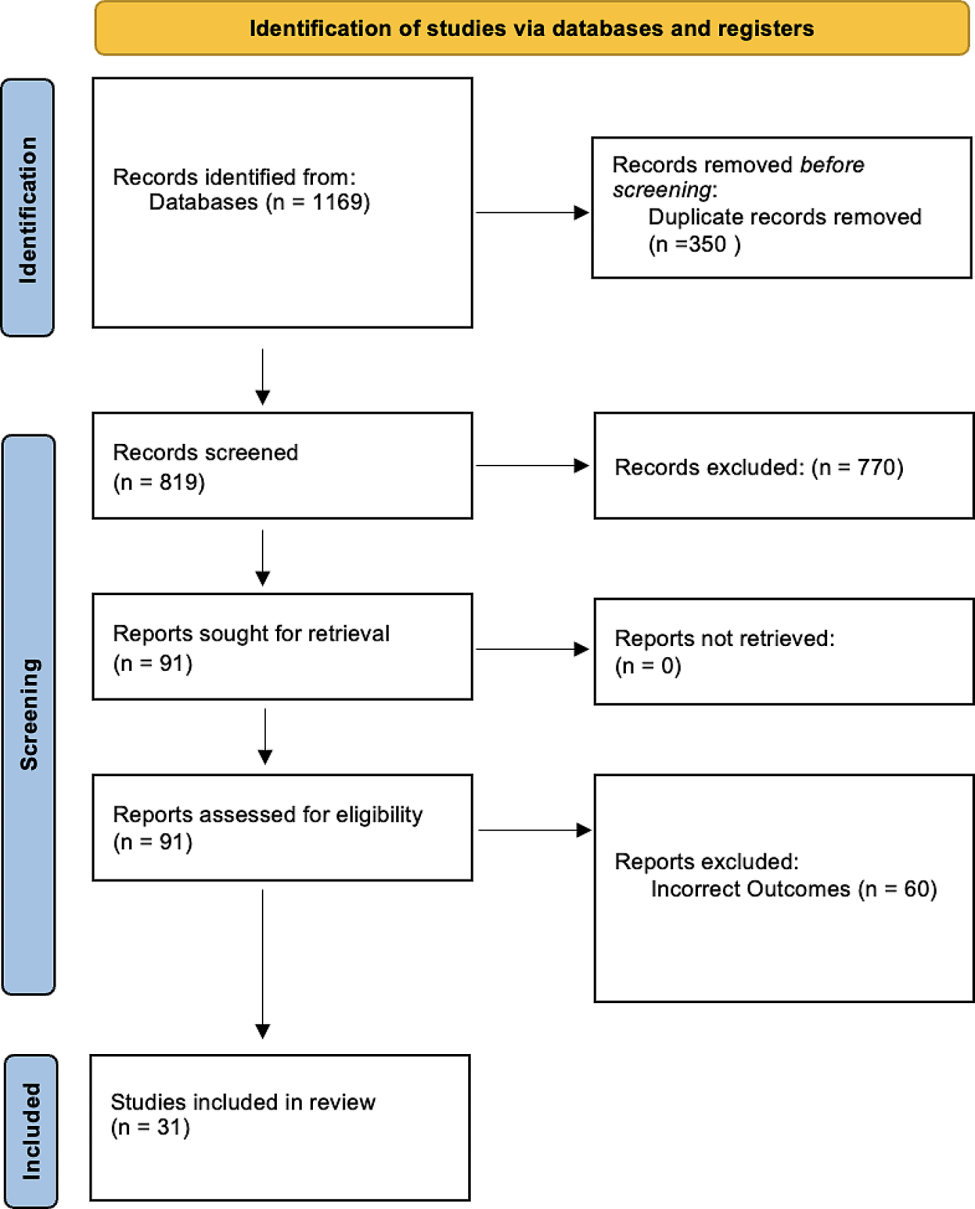
Prisma diagram demonstrating included studies

In order to identify articles related to ICI-mediated pericarditis, a comprehensive search of the databases: Ovid Embase, Ovid Medline, Cochrane Register of Clinical Trials, and Web of Science were completed on May 16th, 2023. Overall, we focused our search on results from 2010 to present. A total of 1169 citations were uploaded into Covidence, the software program used to manage the screening process. After Covidence removed 350 exact duplicates, 819 citations remained for title & abstract screening. Eventually, 31 studies were included in final analysis. Two independent researchers (AK, AA) assessed and screened data in a method consistent with the Preferred Reporting Items for Systematic Reviews and Meta-Analyses guidelines (PRISMA) (Fig. 1) [4]. Conflict resolution during the screening process was undertaken by another reviewer (DR). Letters to the editors, review articles, animal studies, populations including pediatric patients, and articles in languages other than English were excluded.

We employed the patient intervention, control, and outcome framework in our qualitative systematic. Our inclusion criteria included patients > 18 years old who presented with ICI mediated pericardial disease. The intervention employed was medical *±* surgical therapy. The outcomes were development of cardiac tamponade, morbidity, and mortality. To our knowledge, no observational studies have been done previously, therefore, we did not include any comparison groups for analysis.

Included data was compiled for analysis. We did not include a comparison group given the nature of the study. Categorical variables were described using proportions (%). Continuous variables were described with mean or median. Statistical analysis was completed via SPSS 23.0 software (IBM Corp., Armonk, New York).

## Results

### Epidemiology/clinical characteristics

In total, 31 cases encompassing 38 patients were included. Most patients were males (*n* = 31, 72%) with a median age of 63 (IQR: 55–69). The majority of cases identified were from the United States of America (*n* = 13, 30%) or Asia (*n* = 11, 25%). The most common presenting symptoms were dyspnea (*n* = 26, 59%), chest pain (*n* = 14, 32%), and bilateral leg edema (*n* = 2, 5%). Electrocardiogram (EKG) findings of pericarditis were present in 8 (19%) patients, whereas 1 (2%) patient presented with a pericardial rub. Concomitant myocardial involvement was noted in 7% (*n* = 3) of patients. Eighteen (41%) patients presented with cardiac tamponade (Table 1).

**Table 1:** Clinical characteristics of the cohort

Demographics	
Gender (M)	31 (72%)
Age (Median)	63 (IQR:56–69)
Country	
United States of America	13 (30%)
Asia	11 (25%)
Europe	6 (16%)
Other	8 (21%)
Symptoms	
Shortness of Breath	26 (59%)
Chest Pain	14 (32%)
Edema	2 (5%)
EKG findings of Pericarditis/Cardiac Tamponade	8 (19%)
Pericardial Rub	1 (2%)
Concomitant Myocardial Involvement	3 (7%)
Initial Presentation of Cardiac Tamponade	18 (41%)
Cancer Type	
Lung Adenocarcinoma	23 (63%)
Lung Squamous Cell Carcinoma	7 (18%)
Melanoma	3 (8%)
Renal Cell Carcinoma	2 (5%)
Other	3 (6%)
Immune Checkpoint Inhibitor Type	
Nivolumab	23 (61%)
Pembrolizumab	13 (34%)
Ipilimumab	4 (10%)
Other	1 (5%)
Prior Chemotherapy	25 (66%)
Prior Radiation/Surgery	13 (35%)
Confirmed Metastasis	17 (39%)
Number of Immune Checkpoint Inhibitor Cycles Prior to Symptom Onset (Median)	4 (IQR:2–6)

The most common cancers necessitating ICI therapy were lung adenocarcinoma (*n* = 23, 63%), lung squamous cell cancer (*n* = 7, 18%), melanoma (*n* = 3, 8%), and renal cell carcinoma (*n* = 2, 5%). The most common ICIs were nivolumab (*n* = 23, 61%), pembrolizumab (*n* = 13, 34%), and ipilimumab (*n* = 4, 10%). The median number of ICI cycles prior to symptom onset was 4 (IQR: 2–6). Prior chemotherapy had been undertaken in 66% (*n* = 25) of patients, while 35% (*n* = 13) had undergone prior radiation (Table 1).

### Imaging

The most frequent finding on Chest X-ray was enlarged cardiac silhouette (*n* = 6, 14%). Computed tomography (CT) findings demonstrated a pericardial effusion in 21 (60%) patients with the predominant size being large (*n* = 10, 25%). A total of 4 (9%) patients underwent cardiac magnetic resonance imaging (CMR) testing. Of these 4, 2 (5%) had delayed hyperenhancement and 1 (2%) had positive T2 short tau inversion recovery. Transthoracic echocardiogram (TTE) was the most common overall modality used for confirmatory diagnosis (*n* = 29, 83%). The most common findings were tamponade (*n* = 13,30%) and large pericardial effusion (*n* = 7, 16%) (Table 2).

**Table 2:** Imaging findings of included cohort

Imaging	
Chest X-Ray Findings	
Cardiac Enlargement	6 (14%)
Computed Tomography Effusion Size	
Undetermined	7 (18%)
Large	10 (25%)
Moderate	2 (5%)
Small	2 (5%)
Transthoracic Echocardiogram Effusion Size	
Undetermined	3 (8%)
Large/Tamponade	22 (75%)
Other	6 (15%)

### Outcomes

Medical management consisted primarily of corticosteroids (*n* = 26, 59%), colchicine (*n* = 8, 18%), and non-steroidal anti-inflammatory drugs (NSAIDs) (*n* = 6, 14%). Pericardiocentesis occurred in 68% (*n* = 26) of patients, whereas pericardial window occurred in 21% (*n* = 9). Median fluid drained was 540 mL (IQR: 400–1000). Cytology was obtained in 66% (*n* = 25) of patients with 25% (*n* = 11) being positive for malignancy. Overall, 35 (92%) patients had symptom improvement with cessation of ICIs. Resumption of ICI occurred in 15 (34%) patients. Of those that resumed ICI therapy, recurrence occurred in 47% (*n* = 7) of patients. Median follow up was 210 (IQR:46–495) days. Overall mortality was 16% (*n* = 7) (Table 3).

**Table 3:** Outcomes data of included cohort

Outcomes	
Pericardiocentesis	26 (68%)
Pericardial Window	9 (21%)
Pericardial Fluid Extraction (Median)	540 mL (IQR: 400–1000)
Cytology Positive for Malignancy	11 (25%)
Medical Management	
Non-Steroidal Anti-Inflammatory Drugs	6 (14%)
Colchicine	8 (18%)
Corticosteroids	26 (59%)
Disease Modifying Anti-Rheumatic Drugs/Biologics	1 (3%)
Symptom Improvement with ICI cessation	35 (92%)
Resumption of ICI	15 (34%)
Recurrence of Symptoms After ICI Resumption	7 (47%)
Follow Up (Median)	210 (IQR: 46–495) days
Mortality	7 (16%)

## Discussion

To our knowledge, this is the most comprehensive analysis of ICI-mediated pericardial disease reported to date.

The incidence of ICI mediated pericardial disease remains unknown. Prior systematic reviews and retrospective studies of patients treated with ICIs have reported varied incidence and prevalence rates [1, 5, 6]. Patients with ICI mediated pericarditis in these studies were often males with lung cancer, and nivolumab and pembrolizumab were the two most common ICIs used [1, 7, 8]. Interestingly, men likely have higher rates of irAEs due to the disparity in ICI treatment in males as compared to females [1, 9]. Similarly, in our cohort, we found ICI-associated pericardial disease predominantly affects males, accounting for 72% of cases, with a median age of 63 years. Lung cancer (adenocarcinoma and squamous cell carcinoma) was the most common cancer in our cohort (81% of cases). In a study of 60 patients with advanced non-small cell lung caner receiving immunotherapy with either nivolumab or pembrolizumab, pericardial effusion was found to be a relatively common complication occurring in 7% of patients [5]. We observed that the PD-1 inhibitors nivolumab and pembrolizumab were the most commonly implicated ICIs, used in 61% and 34% of cases, respectively.

Understanding the timeframe for the development of pericardial effusion after ICI initiation is crucial for timely diagnosis and management. Our study revealed an average time to onset of pericardial effusion after 4 cycles of ICI therapy. Prior studies have reported ranges as wide as 1 to 12 months [1, 8–11]. Sawada et al. reported a case of a 67-year-old man who developed pericardial effusion which resolved with corticosteroids after 94 cycles of Nivolumab [12]. Overall, this suggests that pericarditis may be a risk during any cycle of ICI therapy.

Dyspnea and chest pain were the most reported symptoms in our study. Remarkably, only 19% of patients exhibited ECG abnormalities, and a minority displayed physical exam abnormalities, such as pericardial rub (2%). These findings underscore the occult nature of pericardial disease in cancer patients undergoing immunotherapy. Given the poor sensitivity of ECG and physical examination in detecting ICI-mediated pericardial disease, our findings suggest the necessity of a low threshold for further diagnostic imaging when patients present with symptoms. The wide range of reported incidence in previous studies likely reflects the under-diagnosis of pericardial effusion, especially when limited to cases requiring drainage [8]. 

Currently, there are limited prognostic tools to suggest development of ICI-mediated pericardial disease, our findings highlight the importance of continued surveillance throughout the course of treatment as there can be a significant delay from therapy initiation and the development of pericardial disease [13]. Current data suggests baseline EKG and serial troponin measurement to monitor for development [14–16]. 

Despite the assistance of imaging, the diagnosis of ICI-mediated pericardial disease is complex. Causes of effusion including cancer progression, pseudo progression, or infection are other possibilities in this patient population complicating appropriate diagnosis [17]. Progression and pseudo progression can be particularly difficult to distinguish from ICI-mediated pericarditis, as patients may present with effusion and imaging evidence of tumor pseudo-growth due to inflammation from response to therapy [18]. 

Interestingly, of 25 patients who underwent cytological testing of their effusions, 11 tested positive for malignant cells. Similarly, Gong et al. reported 8 of 15 patients who underwent pericardiocentesis for ICI mediated pericardial effusion had malignant cells present on cytology [11]. This finding suggests that pericardial effusion due to ICI may have malignant cells, and that consideration of the overall clinical picture is necessary for accurate diagnosis and differentiation from the similar but distinct entities of malignant pericardial effusion and pseudo progression.

A substantial portion of patients in our study, 68%, required pericardiocentesis, with 41% experiencing cardiac tamponade. These rates mirror those reported in prior studies, underscoring the significant morbidity and mortality associated with pericardial effusion in ICI-treated patients [17]. Almost all patients, 92%, had symptomatic improvement with cessation of ICI. ICI therapy was resumed in 38% of patients, and half of these patients experienced recurrence of pericardial effusion. Over a median follow up of 210 days, the mortality rate amongst patients with pericardial effusion was 16%. Gong et al. found that patients treated with ICI who developed pericardial effusion had an increased risk of mortality (Hazard Ratio: 1.53) compared with those who did not [11]. Thus, the development of ICI mediated pericardial effusion may be a poor prognostic factor for survival [19].

Despite our best efforts, our review is not without several limitations. The entirety of our cohort was derived from case reports with inherent publication bias present. Our analysis may overestimate the severity of this condition as severe case are more likely to be reported. Additionally, given the lack of a control group, our results have limited generalizability.

## Conclusion

ICIs are a novel therapy used for the management of several malignancies. While they are effective for treatment of malignancy, they possess certain cardiotoxic side effects including development of pericardial disease. Patients with ICI mediated pericarditis often present in life threatening cardiac tamponade and definitive diagnosis requires a combination of imaging and pericardial fluid analysis. Management is primarily cessation of ICI therapy coupled with a combination of NSAIDs, colchicine, and steroids. Overall if diagnosed early, mortality rates are low.

### Electronic supplementary material

Below is the link to the electronic supplementary material.

Supplementary Material 1: Supplement: Supplemental References

## Data Availability

Available upon reasonable request.

## Abbreviations

ICI: Immune checkpoint inhibitors
TTE: transthoracic echocardiogram
CT: computed tomography
