# Supplementary material for: Immune checkpoint inhibitors and pericardial disease: a systematic review
Source: Cardiooncology. 2024 May 17;10:29. doi: 10.1186/s40959-024-00234-0 (PMC11100143; doi:10.1186/s40959-024-00234-0)
Supplement: Supplementary file 1 — Supplementary Material 1: Supplement: Supplemental References [file 40959_2024_234_MOESM1_ESM.docx]

Supplemental References:

1. Kushnir I, Wolf I. Nivolumab-Induced Pericardial Tamponade: A Case Report and Discussion. *Cardiology*. 2017;136(1):49-51. doi:10.1159/000447053

2. Kodani M, Yamaguchi K, Sakamoto T, et al. P49-6 Two cases of unexpected cardiac tamponade as pseudoprogression due to immune checkpoint inhibitor in patients with advanced lung adenocarcinoma. *Ann Oncol*. 2021;32:S356. doi:10.1016/j.annonc.2021.05.772

3. Verhaert M, Mebis J, Aspeslagh S, von Kemp B. Steroid-dependent pericarditis following anti-PD1 immunotherapy in a metastatic melanoma patient: a case report. *Eur Heart J Case Rep*. 2023;7(3):ytad112. doi:10.1093/ehjcr/ytad112

4. Vatsala S, Vasireddy S, Krueger SA. Rapidly-Occuring Cardiac Tamponade Due to Immune Checkpoint Inhibitor Use in a Patient with Mesothelioma. *J Am Coll Cardiol*. 2023;81(8):3591. doi:10.1016/S0735-1097(23)04035-4

5. Patel K, Trivedi K, Suleiman A. Pericarditis in a Patient on Pembrolizumab. *Chest*. 2022;161(6):A82. doi:10.1016/j.chest.2021.12.114

6. Zarogoulidis P, Chinelis P, Athanasiadou A, et al. Possible adverse effects of immunotherapy in non-small cell lung cancer; treatment and follow-up of three cases. *Respir Med Case Rep*. 2017;22:101-105. doi:10.1016/j.rmcr.2017.07.004

7. Sawada R, Matsui Y, Uchino J, et al. Late-onset Pleural and Pericardial Effusion as Immune-related Adverse Events after 94 Cycles of Nivolumab. *Intern Med Tokyo Jpn*. 2021;60(22):3585-3588. doi:10.2169/internalmedicine.7219-21

8. de Almeida DVP, Gomes JR, Haddad FJ, Buzaid AC. Immune-mediated Pericarditis With Pericardial Tamponade During Nivolumab Therapy. *J Immunother Hagerstown Md 1997*. 2018;41(7):329-331. doi:10.1097/CJI.0000000000000217

9. Ng P, Ong CC, Tan LL. An Unusual Case of Pericarditis. *Acta Cardiol Sin*. 2021;37(6):648-651. doi:10.6515/ACS.202111_37(6).20210531A

10. Saade A, Mansuet-Lupo A, Arrondeau J, et al. Pericardial effusion under nivolumab: case-reports and review of the literature. *J Immunother Cancer*. 2019;7(1):266. doi:10.1186/s40425-019-0760-4

11. Sakai T, Sasada S, Jyo C, Ishioka K, Takahashi S, Nakamura M. Acute myocarditis and pericarditis after nivolumab treatment in patients with non-small cell lung cancer. *Ann Oncol*. 2017;28:ix90. doi:10.1093/annonc/mdx697.072

12. Yun S, Vincelette ND, Mansour I, Hariri D, Motamed S. Late onset ipilimumab-induced pericarditis and pericardial effusion: a rare but life threatening complication. *Case Rep Oncol Med*. 2015;2015:794842. doi:10.1155/2015/794842

13. Ali MR, Darwish OJ, Alhuneafat L, Abdallah BN, Saleh Y. Rechallenging nivolumab following immune checkpoint inhibitor-induced pericarditis. *Proc Bayl Univ Med Cent*. 2023;36(1):83-84. doi:10.1080/08998280.2022.2132367

14. Harada K, Ogasawara M, Shido A, et al. Pericardial tamponade during pembrolizumab treatment in a patient with advanced lung adenocarcinoma: A case report and review of the literature. *Thorac Cancer*. 2020;11(5):1350-1353. doi:10.1111/1759-7714.13399

15. Itani W, Austin A, McCarthy L, Neu K. Loculated Pericardial Effusion Secondary to Pembrolizumab. *Chest*. 2019;156(4):A664. doi:10.1016/j.chest.2019.08.645

16. Braden J, Lee JH. Immune Checkpoint Inhibitor Induced Pericarditis and Encephalitis in a Patient Treated With Ipilimumab and Nivolumab for Metastatic Melanoma: A Case Report and Review of the Literature. *Front Oncol*. 2021;11:749834. doi:10.3389/fonc.2021.749834

17. Pollock J, Castillo E. Diagnosis and Management of Pembrolizumab-Associated Pericardial Effusion in a Non-small Cell Lung Cancer Patient. *Cureus*. 2023;15(4):e37556. doi:10.7759/cureus.37556

18. Saji AM, Venkataramanan SVA, Hadley ML. Colchicine Isn’t Always The Answer! *J Am Coll Cardiol*. 2022;79(9):2725. doi:10.1016/S0735-1097(22)03716-0

19. Kolla BC, Patel MR. Recurrent pleural effusions and cardiac tamponade as possible manifestations of pseudoprogression associated with nivolumab therapy- a report of two cases. *J Immunother Cancer*. 2016;4:80. doi:10.1186/s40425-016-0185-2

20. Uczkowski D, Ashraf H, Sekhri A, Samad A. Pembrolizumab induced pericardial tamponade: A case report. *Clin Case Rep*. 2023;11(5):e7298. doi:10.1002/ccr3.7298

21. Vittorio A, Sharma R, Siejka D, Bhattarai K, Hardikar A. Recurrent Pericardial Effusion While Receiving Nivolumab for Metastatic Lung Adenocarcinoma: Case Report and Review of the Literature. *Clin Lung Cancer*. 2018;19(5):e717-e720. doi:10.1016/j.cllc.2018.05.010

22. Malhotra G, Chick JFB, Srinivasa RN, Hussain JS, Gemmete JJ, Srinivasa RN. Mediastinal Lymphangioma Complicated by Chylopericardium, Tamponade, and Cardiac Arrest Treated with an Endolymphatic Stent Graft. *J Vasc Interv Radiol*. 2018;29(10):1438-1439. doi:10.1016/j.jvir.2018.03.017

23. Yamasaki M, Daido W, Saito N, et al. Pericardial Effusion With Tamponade in Lung Cancer Patients During Treatment With Nivolumab: A Report of Two Cases. *Front Oncol*. 2019;9:4. doi:10.3389/fonc.2019.00004

24. Chye AM, Nordman IIC, Sverdlov AL. Successful immune checkpoint inhibitor rechallenge after immune-related pericarditis: Clinical case series. *Front Cardiovasc Med*. 2022;9:964324. doi:10.3389/fcvm.2022.964324

25. Tachihara M, Yamamoto M, Yumura M, Yoshizaki A, Kobayashi K, Nishimura Y. Non-parallel anti-tumour effects of pembrolizumab: a case of cardial tamponade. *Respirol Case Rep*. 2019;7(3):e00404. doi:10.1002/rcr2.404

26. Atallah-Yunes SA, Kadado AJ, Soe MH. Pericardial effusion due to pembrolizumab-induced immunotoxicity: A case report and literature review. *Curr Probl Cancer*. 2019;43(5):504-510. doi:10.1016/j.currproblcancer.2019.01.001

27. Benjamin L, Jean-Charles G, Laurence M, Adrien R, Terry L, Régis D. Malignant pericardial effusion complicated by cardiac tamponade under atezolizumab. *SAGE Open Med Case Rep*. 2021;9:2050313X211036005. doi:10.1177/2050313X211036005

28. Khan AM, Munir A, Thalody V, Munshi MK, Mehdi S. Cardiac tamponade in a patient with stage IV lung adenocarcinoma treated with pembrolizumab. *Immunotherapy*. 2019;11(18):1533-1540. doi:10.2217/imt-2019-0067

29. Shaheen S, Mirshahidi H, Nagaraj G, Hsueh CT. Conservative management of nivolumab-induced pericardial effusion: a case report and review of literature. *Exp Hematol Oncol*. 2018;7:11. doi:10.1186/s40164-018-0104-y

30. Moriyama S, Fukata M, Tatsumoto R, Kono M. Refractory constrictive pericarditis caused by an immune checkpoint inhibitor properly managed with infliximab: a case report. *Eur Heart J Case Rep*. 2021;5(1):ytab002. doi:10.1093/ehjcr/ytab002

31. Asai M, Kato Y, Kawai S, et al. Management of cardiac tamponade during nivolumab of lung cancer with intrapericardial bleomycin: case report. *Immunotherapy*. 2019;11(6):467-472. doi:10.2217/imt-2019-0003
